#  Prolapsus d’une colostomie périnéale pseudo continente: une complication exceptionnelle

**DOI:** 10.11604/pamj.2018.30.258.15294

**Published:** 2018-08-07

**Authors:** Abdelouhab El Marouni, Ahmed Zerhouni, Karim Hassani Ibn Majdoub

**Affiliations:** 1 Service de Chirurgie Viscérale, Département de Chirurgie Digestive et Endocrinienne, Centre Hospitalier Universitaire Hassan II de FES, Maroc

**Keywords:** Colostomie périnéal, pseudocontinente, prolapsus stomial, traitement chirurgical facile, Pseudocontinent perineal colostomy, stomial prolapse, easy surgical treatment

## Abstract

Le prolapsus stomial est une complication exceptionnelle et tardive du prolapsus d'une colostomie périnéale pseudo continente (CPPC). Des rares cas ont été décrits dans la littérature, on vous rapporte l'observation ainsi notre attitude vis-à-vis d'une telle situation ou un patient de 75 ans sans antécédents pathologiques, a été opéré initialement pour un adénocarcinome rectal à 1cm de la marge anale post radio-chimiothérapie et non métastatique. Une amputation abdomino-périnéale a été réalisée par voie cœlioscopique avec confection d'une colostomie pseudo continente (CPC). Au cours de la surveillance de sa maladie cancéreuse, l'examen clinique a objectivé un colon qui était prolabé à travers l'orifice stomial d'où son hospitalisation, pour traitement chirurgicale. Les inconvénients de la CPPC sont principalement liés à la morbidité opératoire propre aux amputations du rectum (infections et éventrations périnéales), aux complications propres aux stomies (nécrose, prolapsus, hernie). Le prolapsus stomial est une complication exceptionnelle du CPC, une simple surveillance et éducation du patient au technique d'irrigation souvent suffisant, en cas de volumineux prolapsus difficilement réductible, gênant le patient et aussi des suintement, un traitement chirurgical s'impose il consiste en une simple résection du segment prolabé par abord péri stomial, des techniques par agrafage du segment prolabé pouvant être réalisées sous simple sédation ont été rapportés. L'amputation abdomino-périnéale est une intervention mutilante et mal perçue par les patients. La CPPC est une technique simple, sûre et reproductible, avec une morbidité relativement faible. Le prolapsus stomial représente une complication rare et facilement gérable.

## Abstract (en)

Stoma prolapse is an exceptional and late complication of pseudocontinent perineal colostomy. Very rare cases have been described in the literature. We here report the case of a 75 year old patient with no particular past medical history as well as our therapeutic approach. The patient initially underwent surgery for non-metastatic rectal adenocarcinoma measuring 1 cm from the anal margin after radiochemotherapy. Laparoscopic abdominoperineal resection was performed with confection of a continent perineal colostomy. During follow up, clinical examination showed colon prolapse through the stoma orifice for which he was admitted to hospital for surgery. The complications of pseudocontinent perineal colostomy are mainly related to operative morbidity due to rectal resection (infections and perineal eventrations), to stoma complications (necrosis, prolapse, herniation). Stoma prolapse is an exceptional complication of continent perineal colostomy. Monitoring and patient education on irrigation technique are usually sufficient. Surgery is necessary in patients with bulky hardly reducible voluminous prolapse, embarrassing the patient and with weeping. Surgery is based on simple peristromal resection of the prolapsed segment. It has been reported that prolapsed segment grafting under simple sedation can be performed. Abdominoperineal resection is a mutilating procedure which is poorly perceived by patients. Pseudocontinent perineal colostomy is a simple safe and reproducible technique associated with a relatively low morbidity. Stoma prolapse is a rare and easily treated complication.

## Introduction

L'amputation abdomino-périnéale est une intervention mutilante et mal perçue par les patients. La colostomie périnéale pseudo-continente (CPPC) est une technique de reconstruction périnéale après AAP [1], pour cancer ano-rectal. Une technique simple [2], sûre reproductible, cependant, elle impose certaines complications spécifiques à la technique, le prolapsus stomial est parmi ces complications, il s'agit d’une complication exceptionnelle et tardive de CPPC et représente 13 à 20%. Le prolapsus muqueux ou total est gênant par le suintement permanent obligeant le malade à se garnir, le diagnostic est surtout clinique au cours de la surveillance de la pathologie cancéreuse, le traitement est simple essentiellement chirurgical.

## Patient et observation

**Figure 1:**
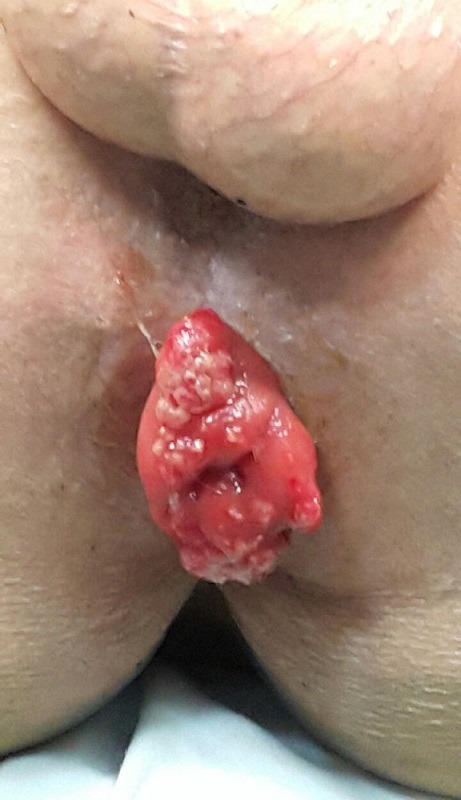
Prolapsus stomial avant le traitement chirurgical

**Figure 2:**
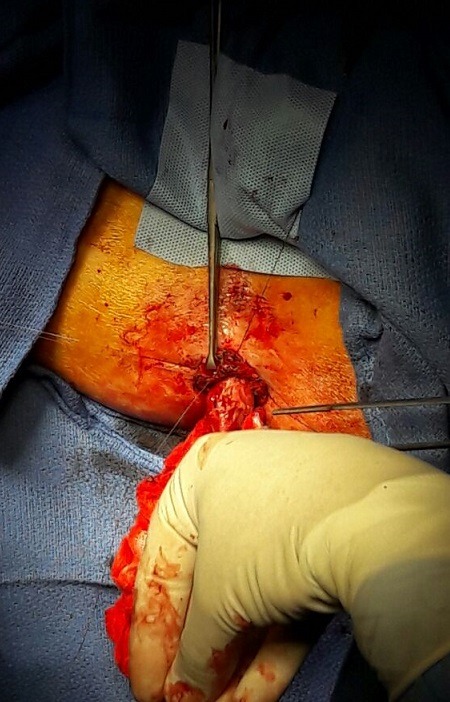
Mucosectomie circulaire avec fixation par des points séparés

Patient de 75 ans, sans antécédents pathologiques, opéré initialement pour un adénocarcinome rectal à 1cm de la marge anale post radio-chimiothérapie et non métastatique. Une amputation abdomino-périnéale a été réalisée par voie cœlioscopique avec confection d'une CPC, la tumeur a été classé pT2N0Mx, et la décision était une surveillance. Le patient était en bon état général avec une bonne évolution. Après irrigation le patient est resté propre toute la journée. Il a été satisfait de sa chirurgie, et il a préféré la CPC à la CIG définitive; l'examen a objectivé (Figure 1) le colon qui est prolabé à travers l'orifice stomial d'où son hospitalisation pour traitement chirurgicale. Le traitement chirurgical a été retenu et le patient a bénéficié de la réalisation d'une mucosectomie circulaire puis une fixation de ce dernier par des points séparés (Figure 2).

## Discussion

**Figure 3:**
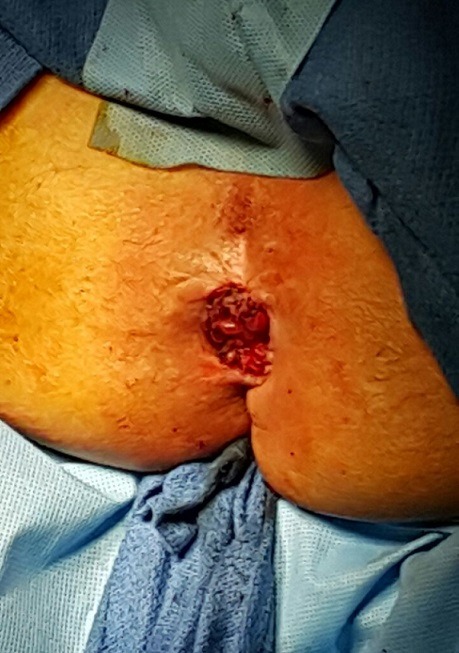
Colostomie pseudo continente après le traitement de prolapsus

Les inconvénients de la CPPC sont principalement liés à la morbidité opératoire propre aux amputations du rectum (infections et éventrations périnéales) [2,3], aux complications propres aux stomies (nécrose, prolapsus, et hernie) Le prolapsus stomial est une complication exceptionnel de la CPC [3], des rares cas ont été décrit dans la littérature, les facteurs de risque sont l'obésité, une augmentation de la pression abdominale, un excès de longueur colique, une simple surveillance et éducation du patient aux techniques d'irrigation souvent suffisant, en cas de volumineux prolapsus difficilement réductible, gênant le patient aussi les suintement, un traitement chirurgical s'impose (Figure 2). Il consiste en une simple résection du segment prolabé par abord péri stomial (Figure 2, Figure 3), des techniques par agrafage du segment prolabé pouvant être réalisées sous simple sédation ont été rapportés [2]. On note l'importance de la colostomie périnéale dans les pays du Maghreb. La colostomie iliaque gauche est toujours source d'inconfort pour nos patients quel que soit leur niveau d'instruction. C'est un concept culturel qui fait que tout colostomisé est considéré comme patient incurable et souvent délaissé par son entourage. Cette situation préoccupe la plupart de nos chirurgiens [4]. Le cas de notre patient illustre l'importance de la surveillance, l'intérêt de l'irrigation pour éviter les autres complications, et la simplicité de la prise en charge (Figure 2) et la qualité [5].

## Conclusion

La CPC est une technique fiable [1]. Son intérêt est considérable dans notre contexte où il peut être proposé en alternative a une colostomie iliaque gauche définitive, qui est souvent mal toléré, après une amputation pour cancer rectal, néanmoins certains impératifs, devraient être respectés: choix minutieux des patients, consentements éclairés, technique chirurgicale impeccable et irrigation colique quotidien définitive [4].

## Conflits d’intérêts

Les auteurs ne déclarent aucun conflits d'intérêts.
